# Systems Connected to Inequities in Access to Kidney Transplantation and the Value of Intersectionality

**DOI:** 10.3389/ti.2024.11658

**Published:** 2024-01-26

**Authors:** Camilla W. Nonterah

**Affiliations:** ^1^Department of Psychology, University of Richmond, Richmond, VA, United States; ^2^Department of Psychiatry, Virginia Commonwealth University Health System, Richmond, VA, United States

**Keywords:** intersectionality, inequities, transplant access, kidney transplant, health disparities

## Abstract

Patients from minoritized backgrounds based on race/ethnicity, gender, sexuality, and other social identities are more likely to experience inequities in access in kidney transplantation. Although these inequities have been reported over the decades, limited research focuses on the experiences of patients with intersecting minoritized social statuses and the mechanisms that contribute to their reduced access to transplantation. Intersectionality, a framework for understanding the ways in which multiple social identities represent interacting systems of oppression and privilege, offers a nuanced approach for understanding the experiences of patients diagnosed with end-stage organ disease with intersecting social identities. This article outlines complex systems that perpetuate inequities by highlighting the value of intersectionality in studying disparate outcomes to transplant and providing recommendations for the transplant community. This article aligns with the ESOT call for action to promote equity in transplantation worldwide.

## Background

Global estimates for Chronic Kidney Disease (CKD) indicate that approximately 10% of the population have a known diagnosis, with diabetes mellitus and hypertension discovered as significant contributors to the development of this condition. This is particularly concerning given high mortality rates associated with the disease [1]. In the US in 2020, it was estimated that 807,920 people had End-Stage Kidney Disease (ESKD). The majority of patients used hemodialysis as their primary form of renal replacement therapy relative to peritoneal dialysis or transplantation [2]. Approximately 30,000 people in the UK were reported as receiving dialysis in 2023, with a yearly rate of 3,000 transplant recipients [3]. Inequities in access to renal transplantation, the optimal form of renal replacement therapy, have been documented for decades [4–8]. These imbalances have been associated with sociocultural factors such as gender, race/ethnicity, income status, and immigrant status. These elements of a patient’s identity often interact with biological and psychological factors resulting in barriers to accessing care [4, 9–13]. Hurdles occur along the steps to transplant, such as referral to transplant, pre-transplant evaluation, being waitlisted, and successful receipt of a deceased or live donor transplant [5, 14]. Insurance issues, financial constraints, limited health literacy, obesity, lack of referrals, reports of discrimination are examples of typical obstacles faced by patients diagnosed with ESKD who seek a transplant [5, 7, 13, 14]. Many patients encounter multiple barriers that interact with each other to affect their successful access to transplantation. This underscores the importance of identifying underlying mechanisms associated with inequities in access to transplantation. The objective of this viewpoint is to highlight complex systems that perpetuate these inequities, discuss the role of intersectionality in examining disparate outcomes to transplant, and provide recommendations to the transplant community. Table 1 provides a list of some key terms and definitions used in the article.

**TABLE 1:** Key terms and definitions.

**Term**	**Definition**
Allostatic load	Cumulative long-term effects of exposure to chronic stress and its impact on an individual’s physical and psychological health
Cultural competence	The ability to understand, value, and respect people’s culture, customs, and belief systems as well as how they vary among individuals or groups
Cultural humility	An ongoing process and commitment to self-reflection of one’s beliefs and assumptions, and an openness to learning from others. It also entails a recognition of power differentials between individuals due to privilege and oppression
Cultural safety	Creating and cultivating an environment that values the cultural background and physical, social, and emotional safety of others. It involves an awareness of one’s personal culture and the ways in which this interacts with the culture of others
Cisgender	A person’s whose gender identity matches their sex assigned at birth. For example, an individual who was assigned female at birth and self-identifies and lives as a woman
Interpersonal racism	Discrimination or bias towards an individual based on their skin tone and/or hair texture. This form of racism occurs between individuals and can be intentional or unintentional
Implicit bias	Having negative beliefs or assumptions about a social group that one is not consciously aware of
Gender identity	One’s personal conception or sense of who they are in relation to their gender. Some people may self-identify as a man, woman, a combination of both, neither, or somewhere along the gender spectrum
Microaggressions	Daily verbal and nonverbal slights, insults, or invalidations that are intentional or unintentional and communicate prejudice towards a person from a specific social group. For example, refusing to call a transgender man by his preferred pronoun, him and instead insisting on using her
Personal pronouns	This is a way of referring to an individual without their actual name. Using the appropriate pronouns for an individual shows respect for their gender identity regardless of what your assumptions may be about them
Socioecological models of health	This model proposes that health is an interaction between multiple environments of an individual such as the person’s individual beliefs or personal characteristics (intrapersonal), exchanges with family, friends, and other support systems (interpersonal), organizations (e.g., health care systems, workplaces), and other systems. All of these different aspects of an individual’s social environment influence their health behaviors and outcomes
Structural racism	Societal expectations, laws, and systems that disadvantage or discriminate against certain racial/ethnic groups, thereby limiting their access to resources such as education, employment, healthcare, and housing
Transgender	A general term for individuals whose gender identity, expression, or behaviors are different from the prototype of the sex they were assigned at birth. For example, an individual who was assigned female at birth but self-identifies and lives as a man

## Sources of Inequities Along the Steps to Transplant

Multiple theories have been implicated in the quest to identify the causes of inequities in access to renal transplantation. Socioecological models account for the interaction between multiple aspects of the individual, for example, at the intrapersonal, interpersonal, institutional, and societal level to affect health outcomes [15, 16]. This model has been used to examine disparities along the steps to kidney transplantation [7, 17]. The role of social determinants of health (i.e., non-medical aspects of an individual’s life such as where they live, are raised, engage in recreational activities, and their vocation) has been established in the literature. Given that these determinants of health overlap with each other as well as biological, sociocultural and political factors, it is important that researchers interrogate these issues and the extent to which they contribute to health inequities.

### Race

In the United States, the origin of many health inequities has been connected to race and racism. Purnell et al. [18] provide examples of the manifestation of racism in the transplant process, specifically for patients with African ancestry who identify as Black. For instance, institutional racism (i.e., implicit or explicit discriminatory practices within the field of transplantation) may be displayed in cultural differences in communication, resulting in provider perception of a patient as non-compliant or uninterested in pursuing transplantation [18]. Race-based modifications for estimating glomerular filtration rate (e-GFR) for Black patients is another example, especially as the removal of these practices indicate positive outcomes for reducing bias [19–21].

### Gender

Transplant data indicates that women are underrepresented in referrals for transplant, more likely to be living donors, less likely to be recruited for clinical trials that investigate immunosuppression and rejection outcomes or receive a transplant [22–25]. Data from the United Network for Organ Sharing and Eurotransplant show disparate outcomes for waitlisting, with women being underrepresented on both kidney waitlists [9].

### Socioeconomic Status

Socioeconomic status has been continuously documented as a barrier to transplant which may be symbolic of classism [4, 5]. Evidence of the role of classism may be especially prominent in countries with universal healthcare or when federally funded programs are offered to patients with ESKD as they continue to experience barriers [8, 26]. Acknowledging that these systems of oppression contribute to inequities in access to transplantation is the first step towards parity and should be an integral part of the work conducted within the transplant community.

## The Importance of Identifying Underlying Mechanisms

An individual’s minoritized status based on race, gender, class, and/or sexuality among others has been connected to physiological and psychological outcomes. One’s social identity can be accompanied with stressors when this identity is disenfranchised in any form, preventing the individual from receiving social benefits afforded to others. As an example, people of European descent tend to have more social benefits and power relative people of African or Asian descent. Race-related stress, a consequence of one’s minoritized status has been connected to persistent occurrences of racism and microaggressions that tax the body’s stress response, resulting in an increase in allostatic load [27, 28]. This rise in allostatic load (refer to Table 1) is linked with a sequela of conditions such as hypertension, depression, and kidney dysfunction, in the absence of adaptive coping approaches to buffer against these stressors [27, 29, 30]. The minority stress model is another example of a theory that has been used to explain the ways in which one’s minoritized status affects their health outcomes. This theory postulates that stigma, prejudice, and discrimination foster stressful social environments for people with a minority status based on their race/ethnicity, gender, and sexuality. This can be compounded by socioeconomic stressors due to poverty, to produce poor mental health outcomes [31, 32]. Concerns about discrimination and risks for depression and anxiety have been documented among transgender and gender non-conforming individuals seeking pharmaceutical care, thereby affecting their health and treatment outcomes [33]. Given the evidence of racism, sexism, transphobia, and other systems of oppression in medicine and how they propagate inequities, ignoring these issues would be a severe disservice to patients with ESKD and other organ diseases.

Social justice initiatives provide a means for empowering transplant professionals and patients invested in health equity. This is in fact an essential part of health equity work if we are to tackle the underlying systems and structures behind these differences rather than proximal estimates such as race/ethnicity, sex, and income status [34]. As the world becomes more diverse through globalization and immigration, the field of transplantation has to evolve to recognize the value of diversity in science. The demographics in the US have changed significantly over the last few decades, a trend that is expected to continue, with more people identifying as Asian, Hispanic/Latine/x or multiracial [35]. Similar trends are apparent in the UK with an increase in the number of people who identify as Asian or Black [36]. Such changes in demographics are accompanied with a multitude of cultural experiences and practices as well as linguistic diversity which must be considered to practice good science [7, 37]. To better support patients, we cannot continue to use color-blind ideological approaches centered on Eurocentric, heteronormative (i.e., perception of heterosexuality as normal and standard), and cisnormative (refer to Table 1) values [37]. This necessitates an all-encompassing transplant science. Decreasing inequities in access to transplantation also comes with a global societal benefit. Diseases weaken an individual’s ability to showcase their talents and make innovative contributions to our society.

## The Value of Intersectionality

A term generated by Kimberlé Crenshaw, intersectionality posits that multiple social identities such as race, gender, sexuality, and disability status can converge at the microlevel of an individual. This could be presented as interlacing systems of privilege and oppression, such as racism, sexism, homophobia, transphobia, classism, and ableism [38]. Bowleg [39] conveyed the significance of applying an intersectionality framework in public health, noting that research focused solely on women or racial/ethnic minorities disregards the intersection of both identities and does not cater to the multidimensional nature of the term, minority. Intersectionality is proposed as valuable for comprehending structural inequalities that pertain to racism, sexism, classism, and other structures to produce health inequities [40–42]. This framework allows researchers to understand the experiences of patients from minoritized groups from their vantage point and actual social realities [40].

Specific to the field of nephrology and transplantation, advocates for intersectionality highlight benefits for eradicating inequities for lesbian, gay, bisexual, transgender, and queer (LGBTQ+) individuals [42, 43]. Other studies have performed statistical analyses that investigate interactions between factors such as gender and race with no direct reference to employing an intersectionality framework. Findings revealed fewer living donor evaluations for Black women, while White and Latino/Hispanic men finalized their pre-transplant evaluation sooner than their women counterparts [44, 45]. Altogether, the literature signifies a paucity of research on intersectionality in transplantation. When intersectional analyses are conducted without implementing an intersectionality framework, these studies do not assess for roots causes of inequities (e.g., racism, sexism, classism) but instead focus on proxies such as race, gender, and income status. The implementation of an intersectionality framework to address health inequities in access to transplantation could provide a nuanced understanding of the experiences of certain groups. For example, published research in the US indicates that people who identify as Black experience more barriers in access to transplant with some associations to institutional racism. Other intersectional questions to consider include: how do the experiences of Black men and women differ? How does the ethnic background, immigration status, and income of a Black individual play a role in access to transplantation? Given the potential for scientific gains in addressing inequities along the steps to transplant, the transplant community is encouraged to be intentional about executing this framework in research and practice.

## Recommendations for Research and Practice

One of the first steps in executing an intersectionality framework is to forgo the standardization of research on Eurocentric, cisgender, heterosexual men. Although we have made strides in improving the gender distribution of participants for clinical trials and other research methodologies, many of the surveys used in research are limiting [22]. The inclusion of simple demographical questions about ethnicity, sexuality, gender identity, and disability status could provide substantive information and facilitate intersectional analyses relative to focusing on race and sex. Moreover, most studies do not distinguish between biological sex (based on chromosomes and reproductive traits), and gender (socially constructed templates for men and women), making it harder to capture the experiences of people who do not subscribe to the gender binary or those who are intersex [9]. Race, a sociopolitical construct, is often used as an indicator for a biological construct even though genetic ancestry is a better measure. Racial differences in access to renal transplantation have been reported for decades yet little research has focused on varying forms of racism (e.g., interpersonal or institutional racism, microaggressions) accounting for some of these differences [18]. Of note, scholars are encouraged to be thoughtful in their use of social variables such as race and gender. Providing a rationale for using these variables and explicitly stating limitations (e.g., gender differences studied did not account for gender diverse individuals) as well as collecting data central to the research question, is useful for improving our scientific understanding of the role of social identities. This also reduces the exploitation of participants, especially when the questions contain personal and/or confidential information.

When examining intersections between aspects of a person’s identity, it is advisable to use measures that explore the root causes of these differences. The literature consists of a plethora of measures that assess constructs such as homophobia, racism, sexism, and transphobia. Sample measures with good psychometric properties include, The Gendered-Racial Microaggressions Scale for Black Women [46], the Lesbian, Gay, Bisexual, and Transgender People of Color Microaggressions Scale [47], The Expectations of Racism Scale [48–50], Everyday Discrimination Scale [51], The Neosexism Scale [52], and Attitudes toward Transgender Men and Women [53]. Studies aimed at exploring the role of these underlying mechanisms can make use of such measures. Many of the measures have already been validated in health populations, including patients with CKD [29, 54, 55]. Understudied areas such as the role of ableism, ageism, and xenophobia warrant more attention and may offer a wider range of understanding of the complexities associated with a patient’s multiple social statuses. Thom et al. [56] suggest that inequities may be apparent for patients with impaired decision-making (e.g., intellectual disability, cognitive impairment due to a condition) pursuing transplant.

Qualitative and quantitative guidelines for implementing an intersectionality framework such as an investigation of power and inequality already exist in the literature [57–59]. In many cases, a combination of both methodologies (i.e., a mixed-methods approach) could provide a deeper understanding of a research question [60]. Intersectionality workshops are also available via the Intersectionality Training Institute.

**FIGURE 1:**
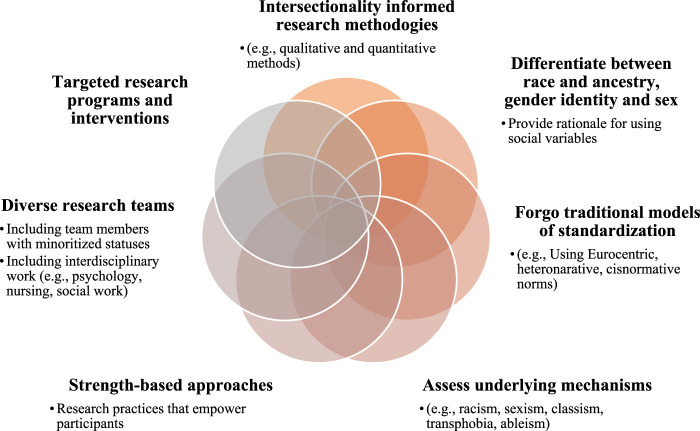
Recommendations for incorporating intersectionality in research.

Strength-based approaches focused on the patient’s strengths and abilities instead of deficiencies can be extremely beneficial. These approaches offer an opportunity for providers to learn from their patients who are the experts of their experiences about the strategies already implemented to navigate the barriers they face. This can also be empowering for patients as they are able to contribute to social justice initiatives related to their health. Community-based participatory research and qualitative research designs are examples of research methodologies that enable patients’ role as co-creators of scientific knowledge. Targeted research questions and interventions (e.g., an intervention focused on Asian immigrant women) will also facilitate in-depth examination of an inequity for a specific group with intersecting identities. Scholars interested in implementing intersectionality in their work should dedicate the time to develop the appropriate proficiency for conducting this work and/or collaborate with other researchers that are already undertaking this work. Diverse research teams with members who possess the minoritized statuses being studied can reduce the potential for enacting additional harms and promote quality research [34]. Awareness of the appropriate terminology such as cisgender versus transgender, personal pronouns, and phrases that may be reflective of microaggressions is crucial to prevent harms on groups that already experience social oppression. Cultivating diverse research teams also promotes social justice given that people from minoritized backgrounds have been reported to receive less extramural funding to support their work in comparison to those from more privileged backgrounds [34]. Interdisciplinary work with professionals from backgrounds such as social work, psychology, and sociology would also strengthen the transplant community’s efforts towards health equity. Figure 1 provides a graphical representation of the different recommendations for research.

Specific to practice, curricula changes are needed to promote a more nuanced understanding of the role of intersectionality in patient care. This includes basic knowledge of the intersectionality framework, and instruction that incorporates clinical case studies with patients with intersecting identities, caution should be taken to ensure that these clinical cases do not perpetuate stereotypes but instead allow trainees to engage in critical thinking about the ways in which systems of oppression disenfranchise certain groups. Sabik [61] developed an intersectionality toolkit for public health education which can be tailed to nephrology populations for pedagogical purposes. To get to the root causes of inequities in transplantation, we cannot focus solely on implicit or unconscious bias training or diversity and inclusion initiatives as many of them fail to empower individuals with the necessary tools to combat bias and oppression [62]. Effective trainings use proactive approaches that empower individuals to identify and dismantle systems that allow injustices to persist. The most efficacious trainings comprehend the value of prioritizing the concerns of the most disenfranchised groups, in addition to cultivating environments that promote safety and belonging [62].

Another aspect of practicing intersectionality is basic knowledge of the sociocultural components of social groups and identities, combined with self-reflection. Some of the terms used to describe this process include cultural competence, cultural humility, and cultural safety [63]. Cultural competence and cultural humility are commonly used within the U.S. whereas cultural safety is typically used in New Zealand, Australia, and Canada [63]. The transplant community is encouraged to consider keys elements of these concepts in their application of any form of culturally relevant care. First, healthcare professionals should cater to the sociocultural needs of the patient group, part of which requires education about the group(s). Second, providers must engage in reflective exercises that entail learning new information and unlearning previously held knowledge, confronting and challenging personal biases and worldviews as well as the ways in which they may conflict or align with other worldviews or experiences. Third, awareness of power structures and differentials that may interfere with the patient-provider relationship and recognition of the ways in which colonization, racism, sexism, and other forms of discrimination contribute to health inequities. Finally, to truly invest in health equity, transplant professionals must recognize that patients are the experts of their own experiences. Therefore, providers should be transparent about their limitations in knowledge and devote themselves to a lifetime of learning [63, 64].

Another extension of administering intersectionality is by expanding the workforce with providers whose identities match those of their patients [65]. This is often the first step in decreasing medical mistrust and fears of discrimination among patients with intersecting minoritized identities. Diversity in transplant personnel improves our aptitude for offering culturally relevant interventions given the personal and professional expertise of these providers. Many associations within the field of transplant such as the American Society of Transplantation (AST) and the European Society for Organ Transplantation (ESOT) have already established a range of diversity initiatives. These include objectives focused on increasing the number of professionals from underrepresented groups and eliminating systemic racism. Other actionable steps could entail advocating for policy changes that mandate medical facilities to report on the demographics of their staff relative to that of their patient populations. Hospitals with complimentary statistics can be incentivized whereas those with little to no diversity can be educated on the significance of enhancing diversity within their institution, including specific instruction on intersectionality. Additionally, healthcare facilities with gaps in diversity could be required to cultivate, for example, a 5-year plan to improve their statistics. As part of their advocacy for policy changes, organizations like the ESOT and AST can outline the multiple health and social benefits of enhanced diversity, including increased revenue and innovation for healthcare systems [66].

**FIGURE 2:**
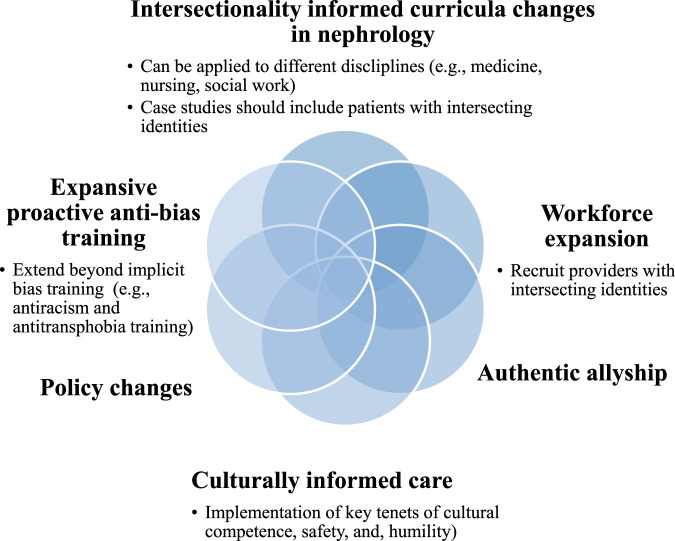
Recommendations for incorporating intersectionality in practice.

Practical efforts towards health equity through an intersectional lens include institutional changes. Examples comprise but are not limited to inclusive in-take forms that account for the reporting of multiple forms of social identities and improved e-GFR guidelines for transgender and gender diverse ESKD patients who have undergone gender affirming hormone therapy. Finally, allyship, is an essential part of health equity and comprises practices and actions undertaken by people with privilege as they advocate for those who are susceptible to systems of oppression. Key tenets of authentic allyship require openness to criticism and constructive feedback, humility, advocacy, and consciousness of one’s power. An essential component of authentic allyship is the amplify the voices of people with marginalized identities without taking up space from those same voices [67]. See Figure 2 for a pictorial view of these recommendations.

## Conclusion

Intersectionality offers a valuable opportunity for the transplant community to make advancements towards parity in access to kidney transplantation. It requires multifaceted approaches and dedicated transplant professionals invested in improving outcomes for all patients.

## Data Availability

The original contributions presented in the study are included in the article/supplementary material, further inquiries can be directed to the corresponding author.
